# VLM-based multi-agent architecture for multifunctional robotic health attendant (RHA) using phase-prompt task design

**DOI:** 10.3389/frobt.2026.1928137

**Published:** 2026-10-29

**Authors:** Kyungki Kim, John Windle, Tom Windle, Melissa Christian, Christian Haas, Bethany Lowndes

**Affiliations:** 1 Durham School of Architectural Engineering and Construction, University of Nebraska-Lincoln, Omaha, NE, United States; 2 Center for Intelligent Healthcare, University of Nebraska Medical Center, Omaha, NE, United States; 3 College of Information Science and Technology, University of Nebraska, Omaha, NE, United States; 4 Department of Neurological Sciences, College of Medicine, University of Nebraska Medical Center, Omaha, NE, United States; 5 Nebraska Medicine, Omaha, NE, United States

**Keywords:** healthcare robotics, model context protocol, multiagent architecture, natural language task design, robotic health attendant, vision language models

## Abstract

**Introduction:**

Healthcare robots are increasingly needed to support clinicians whose work involves diverse tasks and frequent task switching. However, most healthcare robots developed to assist clinicians either perform a single function or implement multiple functions as fixed task pipelines that are difficult to adapt to different patients, rooms, or care teams. This study presents a vision language model (VLM) based multiagent architecture for a multifunctional Robotic Health Attendant (RHA).

**Methods:**

In this architecture, each task can be designed by writing natural language phase specifications that describe how available tools should be used during task execution. A main agent interprets natural language requests from clinicians or patients and routes each request to the appropriate task agent. During execution, the VLM uses language, visual input, and contextual information to select tools and interpret their results. A shared Model Context Protocol (MCP) tool layer provides elemental robot functions such as navigation, manipulation, and perception across tasks. The architecture was implemented on a TIAGo Pro robot and evaluated in an unoccupied patient room across five tasks including object delivery, documentation, visual monitoring, patient education, and medication reminders.

**Results:**

The case study demonstrated the feasibility of the proposed architecture as a reasoning core for a multifunctional healthcare robot by executing multiple tasks and adapting task behavior by editing natural language phase specifications. The comparison across three VLMs showed that longer tool-call sequences led to skipped steps and, in some cases, false reports of task success, which is a key reliability concern for healthcare robots performing long, multi-step tasks.

**Discussion:**

The study also identified technical limitations and future research needs that must be addressed to make the architecture more robust in realistic care settings.

## 1 Introduction

Workforces for health remain under sustained strain worldwide. For example, the World Health Organization (WHO) estimated a global shortage of about 5.8 million nurses in 2023, and shortages of this scale are expected to persist through the end of the decade (World Health Organization, 2025). Nurses face heavy workloads and high rates of burnout, with a survey of more than 43,000 U.S. healthcare workers finding that 56% of nurses reported burnout and 41% intended to leave their job (Rotenstein et al., 2023). This pressure has driven growing interest in service robots that can support clinical staff. A clinician’s shift typically involves many tasks, such as administering medication, assessing patients, documenting care, handling supplies, and communicating with patients and staff, often with frequent switching and interruption (Cornell et al., 2011). Therefore, given the nature of clinicians’ work, reducing this burden would require an intelligent multifunctional robot that can perform many of the tasks they perform, communicate with clinicians and patients in natural language, and let clinicians guide its behavior so that it adapts to the particular state of patients, rooms, and the clinical team’s workflow.

Despite the need for such flexible multifunctional robots in healthcare, most existing systems perform only a single function, such as delivery (Ackerman, 2014; Aethon, 2015; Diligent Robotics, 2023), disinfection (Blue Ocean Robotics, 2025; Xenex Disinfection Services, 2024), patient transport (Baltazar et al., 2021), lifting (Mukai et al., 2010; Ding et al., 2014), or social interaction (Pandey and Gelin, 2018). The fundamental limitation of these systems is that each performs only a narrow part of clinicians’ responsibilities, and robotizing a wider range of work requires the deployment of multiple separate platforms in the healthcare environment. Since operation and interaction in these systems are fixed at development time, clinicians cannot adjust the behaviors of the robots and must instead learn to operate each separate machine, potentially increasing their workload.

There are also robots that perform several functions, such as Hobbit (Bajones et al., 2018), Lio (Miseikis et al., 2020), and HoLLiECares (Schneider et al., 2024), the last of which operates in real hospital trials. These robots meet the need for many functions, yet they share several limitations. First, each task is hard-coded, and clinicians cannot adapt its behavior to a particular room, patient, or care team. Second, the tasks are built individually rather than over a shared layer of common robot skills such as navigation, speech, and listening, which makes the design harder to scale as tasks accumulate. Third, these robots respond through fixed commands and preset routines, without reasoning over the visual, contextual, and verbal information of a situation to understand it and decide what to perform and how.

These limitations stem in part from the tools available when these systems were built, before the ability to develop intelligent agents using language models (LLMs) and vision-language models (VLMs) reached its current capability. In early work, robots were controlled by text-based LLM agents that interpret a natural-language command and determine what the robot should do next. SayCan, built on the PaLM language model, enables a robot to follow an instruction such as “bring me a drink” by selecting an appropriate skill from a predefined set (Ahn et al., 2022). Inner Monologue introduces a feedback loop in which the robot describes what it observes after each step, such as whether an object was found or a grasp succeeded, and returns that description to the model for the next decision (Huang W. et al., 2022). In both systems, the LLM agent does not process the scene directly, and a separate perception module converts what the robot senses into a text description of the scene.

More recent work uses VLMs to develop agents, which receive visual input and the instruction in the same prompt and require no separate conversion of the scene into a text description. These agents have been integrated into robots in different ways. In some approaches, the VLM is used to produce low-level motion directly. PIVOT, for instance, uses a VLM (GPT-4V) to select an appropriate arm motion based on candidate motions rendered on the camera image (Nasiriany et al., 2024). In others, VLM agents make high-level decisions, determining which skill to use in a given situation and the input to that skill. The skills are exposed to the agent as a set of callable tools that the agent can invoke according to its reasoning, a mechanism known as tool calling and standardized through interfaces such as the Model Context Protocol (MCP) (Anthropic, 2024). For example, in BUMBLE, a VLM agent analyzes a natural-language command and the camera view to generate a sequence of up to 12 skills such as navigation and grasping to retrieve objects across the rooms and floors of a building (Shah et al., 2024). These modern VLM-based agent approaches are promising for enabling a multifunctional robot to perform its tasks adaptively in a real care setting, but their use in healthcare robots is still limited.

These advances motivate the VLM-based multi-agent architecture we present for a multifunctional Robotic Health Attendant (RHA), which performs multiple healthcare tasks, each defined to utilize robot skills through a clinician-editable task design written in natural language. Unlike earlier multifunctional robots such as HoLLiECares (Schneider et al., 2024), the architecture proposed in this study uses a VLM for multimodal situational understanding, a multi-agent structure to design tasks in natural language and execute them independently, and MCP to share skills across tasks. In our earlier work, we connected an LLM to a robot for a single task in simulation (Kim K. et al., 2024), and then built an n8n workflow for multiple tasks across physical, communication, and information functions in simulation (Kim et al., 2026). Building on this earlier work, the present study constructs a VLM-based RHA architecture on a real robot. We develop a set of tools for the TIAGo Pro robot and expose them to the agents through MCP. Under a main agent, each task is handled by a separate task agent that clinicians can design and modify in natural language. Finally, we evaluate the system architecture by running five tasks in a patient room across three different VLMs.

This paper makes three contributions. First, we extend recent VLM- and LLM-based agent techniques to a multifunctional healthcare robot, rather than developing a new model or technique. Second, we design the agent architecture so that clinicians can author each task in natural language, using the prompt-based nature of VLM agents that earlier robot systems did not use. Third, we demonstrate the feasibility of the proposed VLM-based architecture for the RHA, where a single architecture performs multiple healthcare tasks on real hardware across different VLMs.

## 2 Related work

### 2.1 Robots for healthcare tasks

Most robots developed for healthcare perform a single function. Mobile platforms such as Moxi (Diligent Robotics, 2023), TUG (Aethon, 2015), HOSPI (Ackerman, 2014), and Relay (Relay Robotics, 2026) deliver medications, specimens, and supplies between hospital units. Ultraviolet-C platforms such as UVD Robots (Blue Ocean Robotics, 2025), LightStrike (Xenex Disinfection Services, 2024) disinfect patient rooms between occupants. Lifting robots such as RoNA (Ding et al., 2014) and RIBA (Mukai et al., 2010) move and reposition patients between bed and chair, and intelligent wheelchairs transport patients through the hospital (Baltazar et al., 2021).

Other systems perform more specialized clinical tasks. Robots draw blood (Giesen et al., 2026), assist patients with eating (Park et al., 2020), and bathe bedridden patients (King et al., 2010). Telepresence platforms such as RP-VITA place a remote physician at the bedside for consultation (Ackerman, 2012). A quadruped platform measures patients’ vital signs at a distance, including skin temperature, respiratory rate, and heart rate (Huang H.-W. et al., 2022).

Some robots support patients through conversation and companionship. Humanoid platforms such as Pepper (Pandey and Gelin, 2018) engage patients through speech and simple interaction, and the seal-shaped PARO provides emotional comfort to older adults and people with dementia (Wada and Shibata, 2007). Companion robots such as ElliQ (Intuition Robotics, 2025), Navel (Navel Robotics, 2020), Robin (Expper Technologies, 2026), and Hyodol (Hyodol, 2020) hold daily conversations and deliver reminders, and recent systems integrate a language model to produce more natural dialogue, as in a socially assistive robot deployed in a geriatric care unit (Blavette et al., 2025). Across these categories, each robot performs one function, and covering a wider part of clinician work requires a separate platform for each task.

A smaller set of robots performs several clinician tasks on one platform. Hobbit performs object pickup, fall detection, reminders, and conversation for older adults at home (Bajones et al., 2018). Lio performs delivery, reminders, exercise guidance, and item handover across care facilities (Miseikis et al., 2020). HoLLiECares performs wheelchair pushing, patient accompaniment, exercise guidance, wound documentation, medicine storage, and deformable-object handling in two hospitals (Schneider et al., 2024). These systems perform several functions on one platform, but they have the common limitations summarized in Table 1. First, the behavior of each task is hard-coded. Hobbit executes each task through a state machine over task-specific scripts (Fischinger et al., 2016; Bajones et al., 2018), Lio executes each task as a behavioral script under a rule-based decision engine (Miseikis et al., 2020), and HoLLiECares (Schneider et al., 2024) uses a separate pipeline for each use case. After deployment, a clinician cannot create a new task or adapt an existing one to a specific patient, room, or care team without a developer modifying the code. This hard-coded design also prevents a clinician from designing or modifying tasks using natural language. Skills such as navigation, speech, and grasping are needed across many care tasks. In these systems, a clinician cannot change the order of skills or how each skill is used within a task. Recent interfaces such as tool calling and MCP support skill composition at the user level through LLMs (Anthropic, 2024). Without an LLM or VLM that provides strong language understanding, these systems interpret user input through fixed keywords or predefined rules (Bajones et al., 2018; Miseikis et al., 2020; Schneider et al., 2024), so a clinician must phrase commands in a preset form instead of their own words. Recent LLMs and VLMs can interpret and understand the context of free-form natural language and map it to robot tasks and actions (Ahn et al., 2022; Huang W. et al., 2022; Shah et al., 2024).

**TABLE 1:** Comparison of multifunctional care robots and the RHA series.

Robot	Task coverage	Control structure	Shared skill layer	Task design/modification by clinician	Natural-language understanding
Hobbit	Pickup, fall detection, reminders, transport, entertainment (5)	State machine, predefined scripts per task	None	Not supported	Command words only
Lio	Delivery, reminders, exercise guidance, handover, disinfection (5)	per-task scripts, rule-based action selection	None	Scheduling and triggering only	Keyword matching
HoLLiECares	Wheelchair pushing, accompaniment, exercise guidance, wound documentation, medicine storage, deformable-object handling (6)	Independently engineered pipeline per task	None	Parameter reset for one task only	Predefined intent matching
RHA #2 (simulation only)	Medication delivery, dialogue, patient education (3)	LLM-orchestrated workflow	Tools called by generated JSON	Natural-language task design	LLM-based
This study (RHA #3)	Delivery, medication reminder, documentation, education, monitoring (5)	VLM agent runtime	Shared tools (MCP)	Natural-language task design	VLM-based

Studies of the authors apply LLMs and VLMs to healthcare robots, referred to as the RHA series. The initial work connects an LLM to a single pick-and-place task in Gazebo simulation (Kim K. et al., 2024). More recent work extends the initial work to multiple tasks, including medication delivery, dialogue, and patient education, in Isaac Sim (Kim et al., 2026). Both studies operate in simulation and use an LLM to produce JSON text from user commands and task specifications written in natural language, which then triggers robot skills. However, they generate the JSON for all skill calls of the entire task at once, instead of checking the outcome of each skill call before the next one. Also, since they use an LLM without native visual understanding, the outcome of visual analysis is reported to the LLM in text form. The study presented in this paper addresses these limitations by utilizing a multimodal VLM as the core for reasoning, creating a multi-agent structure that allows each task to flexibly interpret the user command and task specification to call MCP tools one by one. As in the previous studies, the task descriptions are written in natural language, so a clinician can modify a task with minimal understanding of the robot skills. The following section reviews recent progress in LLM and VLM agents for robots, which advances rapidly outside healthcare and motivates the approach in this study.

### 2.2 LLM/VLM agents for robots

Outside healthcare, recent work uses LLMs and VLMs to control robots through an agent that interprets a command and decides which skill the robot performs next. These agents progress along two connected lines. Early agents use a text-based LLM and read the scene as a text description, while later agents use a VLM and receive the camera image directly.

In the text-based line, the agent reads a command and a text description of the scene and selects an action from a set of robot skills. SayCan runs a mobile manipulator that performs pick, place, and navigation in a kitchen, and it pairs a language model with a value function for each skill (Ahn et al., 2022). The robot holds a fixed set of pretrained skills, each a single unit such as picking up a named object. For each step, the language model scores how well each skill matches the command, the value function scores whether that skill can succeed in the current state, and the product of the two selects the next skill. This design grounds the language model in what the robot can perform, so the agent proposes only feasible actions. However, the language model does not see the scene, every skill is trained in advance, and a skill carries no arguments, so the same skill does not transfer when the target object moves to a new position. Inner Monologue adds a feedback loop to this design (Huang W. et al., 2022). After each step, separate perception models convert the result into text, such as whether an object was found or a grasp succeeded, and the agent reads this text to select the next skill or to revise the plan. This feedback lets the agent recover from a failed step, but the scene still reaches the model only as text produced by separate perception.

A second group within the text-based line lets the language model write a program rather than select one skill at a time. ProgPrompt prompts the model with the available actions and objects written as code, and the model writes the entire plan as a program (Singh et al., 2023). Each action is a predefined function such as a grasp call that takes the target object as an argument, and assertions in the program check that a precondition holds before the action runs. Code as Policies extends this idea from a fixed plan to general control code (Liang et al., 2023). The model writes a program that reads the output of an object detector and passes the detected positions as arguments into control functions, and the program can include loops and arithmetic. In both systems, the model composes calls to predefined functions, which is an early form of an agent that calls tools. The scene still reaches the model through a separate perception module rather than as an image, and the available functions and objects are defined in advance.

The use of VLM removes the text conversion and gives the model the camera image together with the command. A first approach uses the VLM to produce low-level motion directly. Because a VLM returns text rather than continuous coordinates, PIVOT draws candidate motions on the image as numbered options, the VLM selects an option, and the candidates are refined over repeated queries until the motion converges (Nasiriany et al., 2024). MOKA marks candidate key points on the image, the VLM selects the points for a free-form command, and a depth camera converts the selected points into a three-dimensional motion (Fang et al., 2024). Both methods let a VLM control a robot without task-specific training, yet they produce a single low-level motion, which makes long sequences of actions difficult to compose.

A second approach uses the VLM at a higher level, where the agent selects which skill to perform and supplies its arguments. BUMBLE applies a VLM agent to building-wide tasks, such as retrieving an object across rooms and floors, and sequences up to twelve skills in one run (Shah et al., 2024). The agent reads the command and the camera view, selects the next skill, and estimates the skill’s parameters such as the target object, using RGB-D perception and a memory of past steps. This approach performs high-level reasoning over a library of skills and is the closest in structure to the present work. However, it uses a single agent for one type of task, it does not let a user define or modify tasks, and it operates outside healthcare rather than on real hospital hardware.

This progression, from a text-based LLM that selects pretrained skills to a VLM agent that perceives the scene and calls skills at a high level, motivates the architecture in this study. The following section describes how the proposed RHA builds on the VLM-agent approach and addresses the gaps that remain for a multifunctional robot in a real care setting.

### 2.3 Point of departure

Care in a hospital varies across patients, rooms, and care teams, and it changes from day to day. A multifunctional care robot therefore cannot rely on tasks fixed in code, as the robots in Section 2.1 do. It needs a clinician to design and modify each task at the point of care, which requires the natural-language task interpretation of the LLM and VLM agents in Section 2.2. While this agent technology shows clear promise, it does not yet meet the requirements to develop a multifunctional RHA that performs several care tasks and lets a clinician design and modify each task in natural language. Although LLM and VLM agents have advanced rapidly in general robotics, this progress has rarely reached healthcare robots, and no system applies it to a multifunctional RHA. BUMBLE, the closest example, uses a VLM for a single task agent which cannot be applied directly to a multifunctional RHA. The remaining gap is the absence of a tested architecture defining the essential layers of a multifunctional RHA, such as the VLM, the user interface, robot skills shared across tasks, the natural-language task specifications, and the supporting infrastructure for data processing. Given the need for this robot and the promise of this technology, an initial study is urgently needed to design and test the architecture of a multifunctional RHA, providing a reference point for future research in this area.

## 3 Objective and scope

The long-term goal of this research is to develop a robotic health attendant capable of performing the diverse tasks that occupy a clinician’s daily workflow, thereby reducing workload and improving patient care. The objective of this study is to design and evaluate a VLM-based architecture for a multifunctional robotic health attendant that performs multiple healthcare tasks through natural-language task specifications and shared robotic capabilities.

To achieve this objective, we implement the proposed architecture on a TIAGo Pro robot and evaluate it across five healthcare tasks in an unoccupied patient-room environment using three different VLMs. Each task is defined through a natural-language task specification and executed using a shared set of robot capabilities exposed as MCP tools.

The scope of this study is limited to the design and evaluation of the proposed architecture. Human participants are not involved, and clinicians do not create or modify task specifications as part of the evaluation. Furthermore, this work does not seek to improve individual robot capabilities such as navigation, manipulation, perception, or speech interaction. Instead, this study focuses on how a VLM-based architecture organizes and coordinates existing robot capabilities to perform multiple healthcare tasks. Integration with production EHR and clinical documentation systems, as well as operation under unstructured clinical conditions, remains outside the present evaluation.

## 4 System architecture

The proposed architecture follows the standard VLM agent and tool calling pattern. In this pattern, an agent interprets a natural language instruction together with visual or contextual input to perform a task by calling available tools. The MCP provides the interface that exposes tools to the agent, including each tool’s natural-language description, required inputs, and returned fields so that the LLM can determine how to utilize the tool during execution. This study does not introduce a new agent mechanism or a new tool-calling mechanism. Instead, it organizes this existing agent-tool pattern for a multifunctional RHA that can perform multiple healthcare tasks by executing robot tools sequentially according to natural-language task specifications. We refer to this approach, in which each task is specified as a sequence of natural-language phases that direct tool use, as phase-prompt task design.

Figure 1 shows the proposed multiagent architecture consisting of a main agent, task agents, shared MCP tools, and supporting runtime services. The main agent receives a clinician or patient command and invokes the subagent designed for the corresponding task. Each task agent contains natural-language phase descriptions that guide how the task is executed using MCP tools. The MCP tools provide the robot functions used during task execution, and some tools use supporting backend services for vision, speech, retrieval, or scheduling. The architecture separates task design and task execution into two flows. Clinicians define task behavior through natural language phases in the design time flow, and the agents in the robot execute the defined task through MCP tool calls in the runtime flow.

**FIGURE 1:**
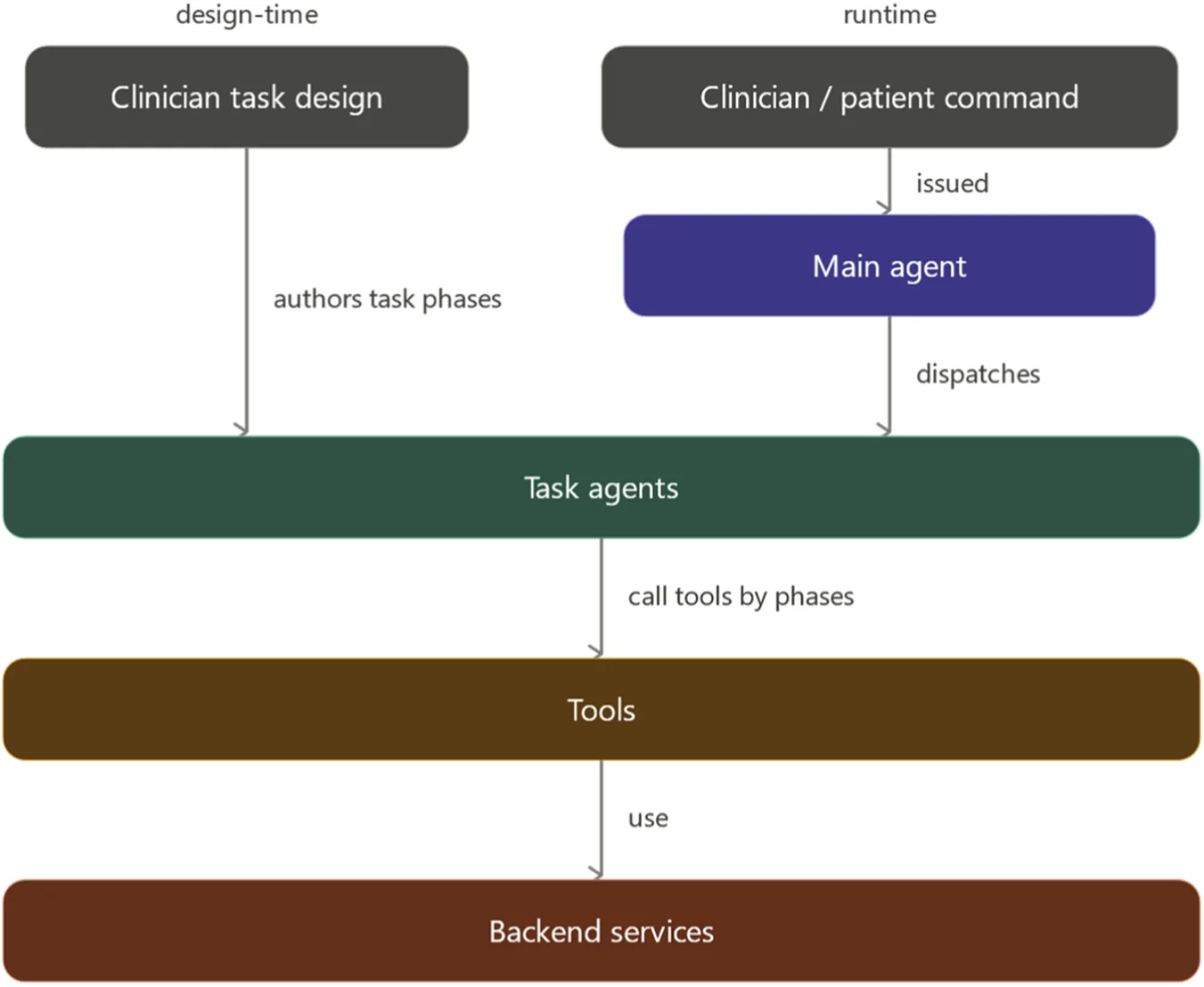
System architecture for multifunctional RHA.

The MCP tools form the shared capability layer within the architecture. They provide individual robot functions that task agents can call during execution. Examples include navigation, rotation, pose adjustment, speech, and UI output. Because the same tool layer is shared across task agents, task agents can use the same robot functionalities combined in different ways with only natural-language instructions in each task agent’s system prompt specifying how the tools should be used.

The supporting backend services are server processes accessed by MCP tools during robot operation. They include vision models, visual grounding, segmentation, speech processing, information retrieval, and scheduling. These services are kept running during robot operation so that MCP tools can access computationally heavy components without repeated initialization. They also allow ongoing processes such as scheduling or monitoring to inform the robot when a timed or event-based input occurs.

In the design time flow, a clinician defines the behavior of a task agent as a sequence of natural language phases. Each phase describes what the robot should accomplish at that stage of the task and how to use one or more tools to complete it. This allows task behavior to be represented as editable text rather than fixed program logic. Because the phase descriptions still need to be written with awareness of the available robot tools, initial task authoring may require support from a developer. However, once the tools and task structure are established, task behavior can be modified by editing the natural language phase descriptions rather than rewriting robot code.

In the runtime flow, the system receives a natural language command together with the visual or contextual information available to the robot. The main agent interprets the command and sends it to the appropriate task agent. The task agent uses its phase descriptions as task specifications and executes the task by calling MCP tools in sequence. At each step, the agent reasons over the phase description, the user command, and the information returned by previous tool calls before deciding the next action. This differs from the earlier studies in RHA development (Kim K. et al., 2024; 2026), where the system generated a sequence of skill calls before execution.

The following section describes how the main agent, task agents, MCP tools, and supporting services were implemented in the RHA system.

## 5 System implementation

### 5.1 System overview

This section describes how the architecture introduced in Section 4 was built with the TIAGo Pro robot and supporting software components. Figure 2 presents the system implementation diagram. At runtime, a command from the patient or clinician interface reaches the main agent, which either dispatches it to one of the five task agents or calls an MCP tool directly. The task agents and the main agent share the same MCP tool layer, and several tools draw on backend services for grounding, segmentation, retrieval, transcription, and scheduling. Each component is described in Sections 5.2 through 5.7, and the five task agents cover the five task types evaluated in Section 6, spanning physical, monitoring, and interactive tasks. The TIAGo Pro robot shown in Figure 3 is the robotic platform for RHA development. The robot has an omnidirectional mobile base, two arms with grippers, and a head mounted RGBD sensor.

**FIGURE 2:**
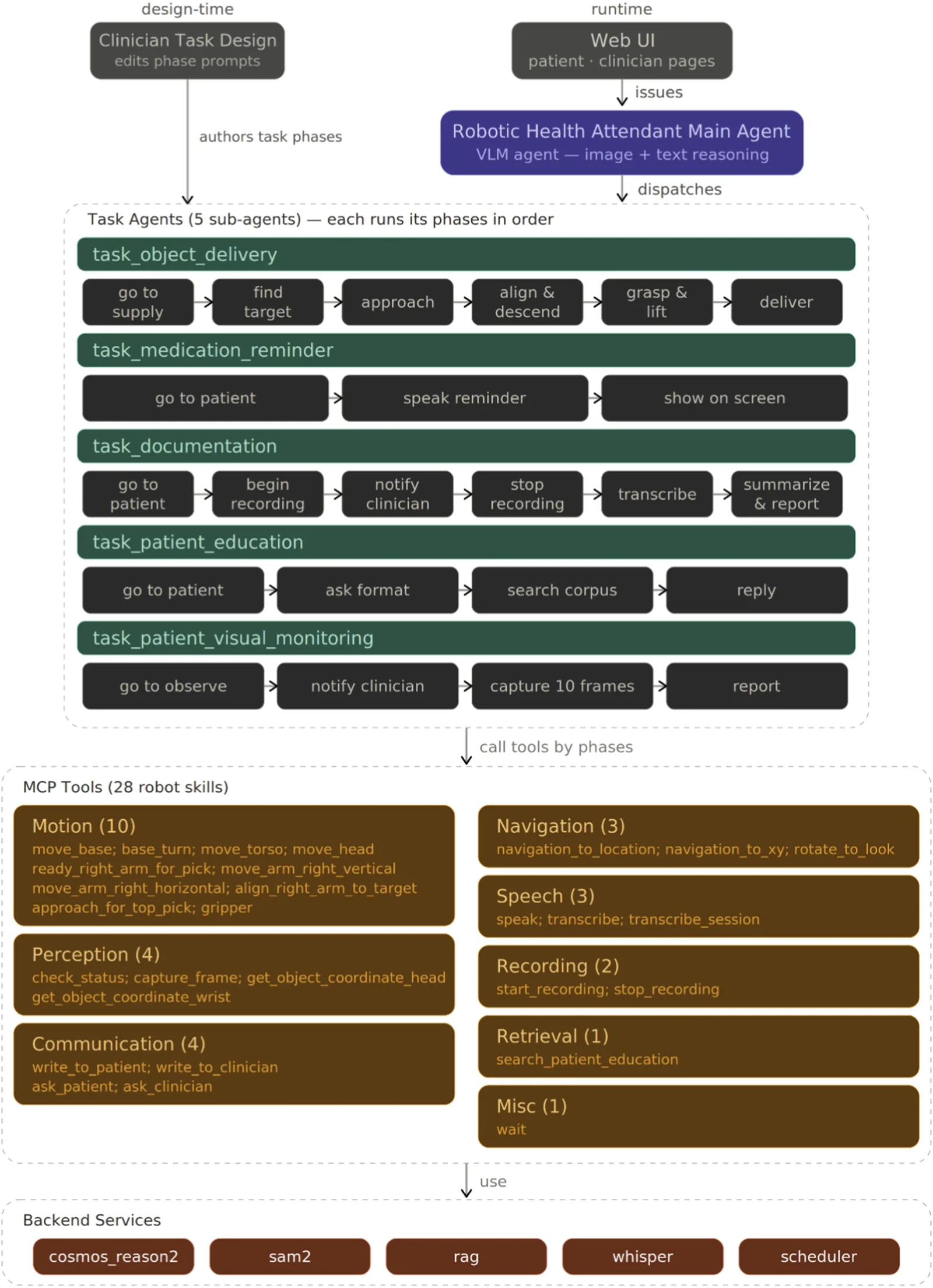
System implementation diagram.

**FIGURE 3:**
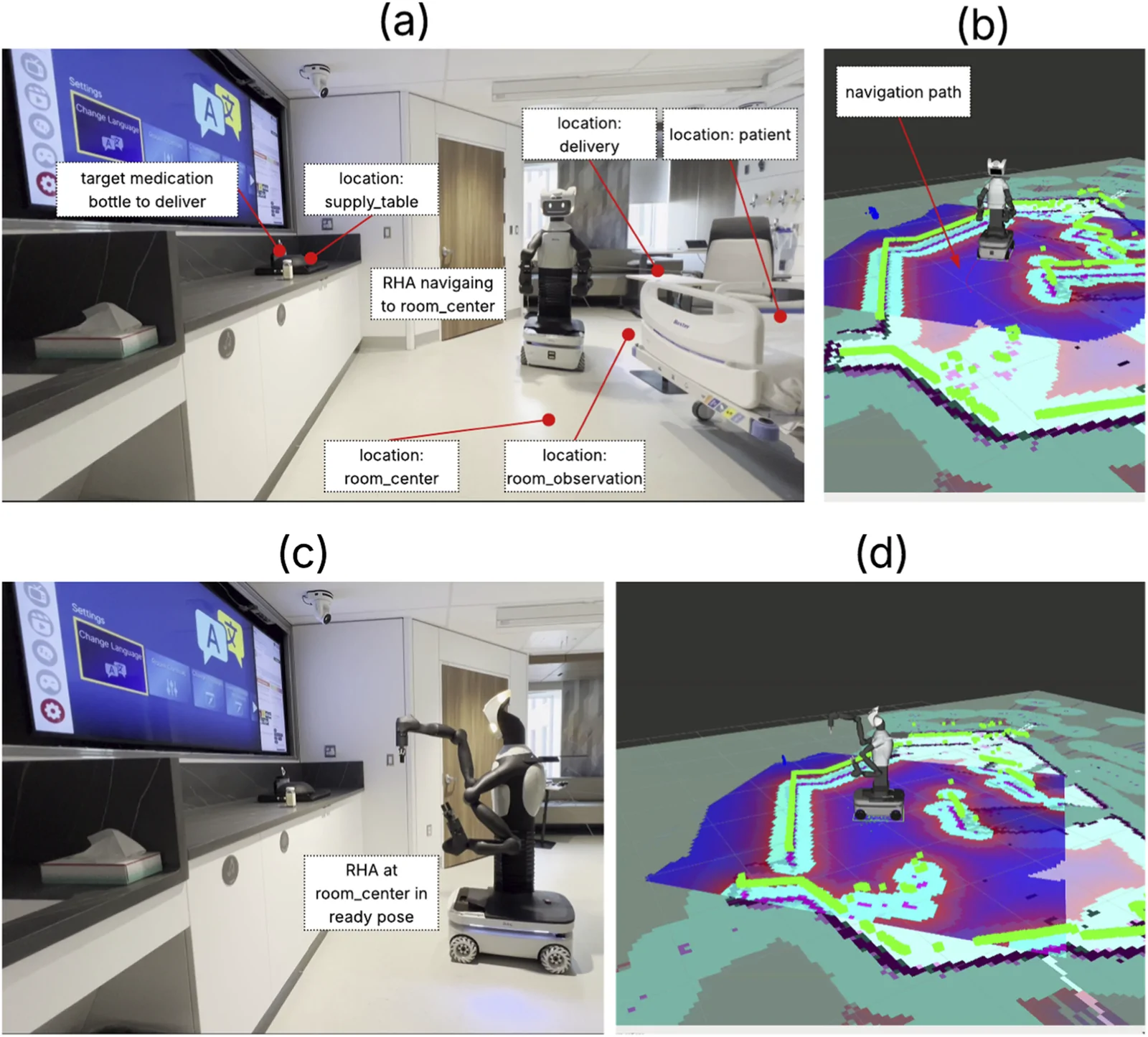
RHA moving to room center, finding the bottle, and preparing the arm (phases 1 to 2 and tool calls 1–5). **(a)** RHA navigating to room_center (tool call 1), with the target medication bottle on the supply table and the saved locations from Table 2 (supply_table, delivery, patient, room_center, room_observation) labeled. **(b)** Navigation path to room_center on the costmap in RViz2. **(c)** RHA at room_center with the right arm raised to the ready pose for the top pick (tool call 5). **(d)** RViz2 view of the RHA at room_center in the ready pose.

### 5.2 VLM-based main agent orchestration

The main agent is the orchestrator of the RHA system. We created a custom provider interface to accommodate different VLM services within the RHA system. This interface formats text messages, image inputs, MCP tool definitions, and tool results into an OpenAI compatible chat completion structure before sending them to the selected model. During development, the main agent used OpenAI gpt-5.4-mini, which is also one of the three models evaluated in the case study. The case study additionally compares two Qwen models (qwen3.5-122b-a10b, qwen3.5-35b-a3b). Across all conditions, the VLM under comparison is the reasoning model that drives the main agent and the task agents, interpreting commands, reasoning over visual input, and selecting MCP tools. All backend services, including the Cosmos Reason2 grounding model and the SAM2 segmentation (Ravi et al., 2024) model used for object localization (Section 5.6), are held fixed across the three conditions. The comparison therefore isolates the agent reasoning model and does not vary the perception backend. The following system prompt defines the main agent’s role and tool-use behavior.

When the runtime process starts, the system loads the system prompt shown in Figure 4, which casts the main agent as the RHA serving both clinicians and patients, and registers all task agents as sub-agent tools combined with the robot skill MCP tools into a single tool list. This gives the main agent two levels of execution. When a user request is a multi-step healthcare task, such as patient monitoring that involves navigation, observation, and reporting, the main agent invokes the matching task agent. When a user request is a quick command, such as moving to a location or speaking a message, the main agent calls an individual robot skill tool directly.

**FIGURE 4:**
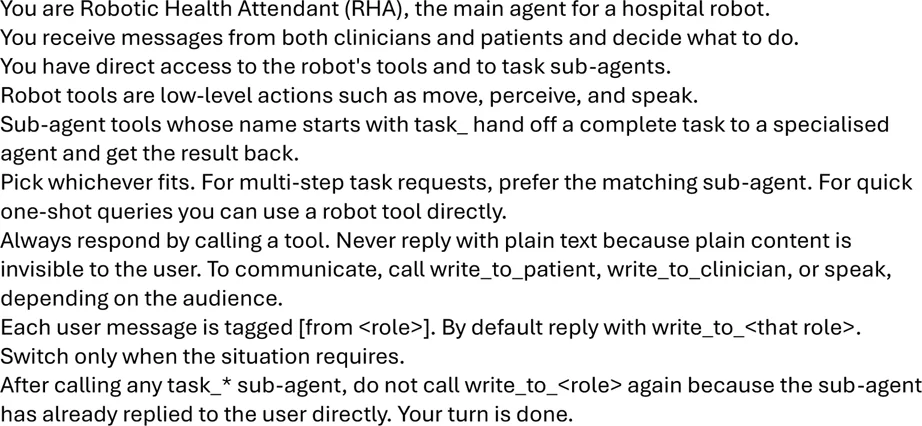
Main agent system prompt.

The task agents are invoked only when the main agent selects them for a matching task request. When the main agent invokes a task agent, it provides the relevant context, including the user input, to the corresponding VLM-based task agent at runtime. The task agent then performs the requested task through its own tool-based execution process and returns a natural-language task result to the main agent, while necessary communication with users is directly handled by specific interaction tools.

The main agent is restricted from responding to users with plain text. Communication with patients and clinicians is performed through interaction tools such as “write_to_patient”, “write_to_clinician”, and “speak”. This makes user interaction an explicit tool-mediated action rather than a plain text response from the agent. Sensor-based perception follows the same tool-mediated structure. When visual information needs to be inspected directly, the main agent or a task agent calls a perception tool such as “check_status” or “capture_frame”, and the returned sensor image is added to the agent context for the next reasoning step. Other perception tools, such as “get_object_coordinate_head”, use backend VLM-based services and return structured perception results instead of inserting the image into the agent context. The same tool-based setup for communication and sensing is used by both the main agent and the task agents.

### 5.3 Shared MCP tool layer

Section 4 introduced MCP tools as the shared capability layer of the architecture. We developed 28 functions as MCP tools that the main agent and the task agents call as shared robot skills. These tools provide elemental robot skills such as navigation, motion, manipulation, perception, and communication. Figure 2 shows the MCP tool layer and its connection to the main agent, task agents, and backend services.

The robot-related MCP tools were developed mostly on top of the TIAGo Pro ROS 2 Humble software stack. We used the ROS 2 Python client library “rclpy” (Macenski et al., 2022) to connect the Python-based MCP agent runtime with ROS 2 actions, services, topics, and robot control interfaces. This allowed each MCP tool to call existing ROS 2 robot capabilities directly from Python, instead of developing each robot capability as a separate compiled ROS package or standalone service.

Each MCP tool is exposed to agents with a tool name, natural language description, input arguments, and returned fields. This exposed information tells the agent what the tool can do, what inputs it must provide, and what result fields it can use after the tool returns. Figure 5 shows how an object localization tool is structured with a natural language description for the agent and the actual robot code that runs when the tool is called. In this example, the tool name “get_object_coordinate_head” and its natural language description allow the agent to understand the tool. The robot side function “locate_object_head” is the code executed when the tool is called, and it should be designed to return the results as described in the tool description.

**FIGURE 5:**
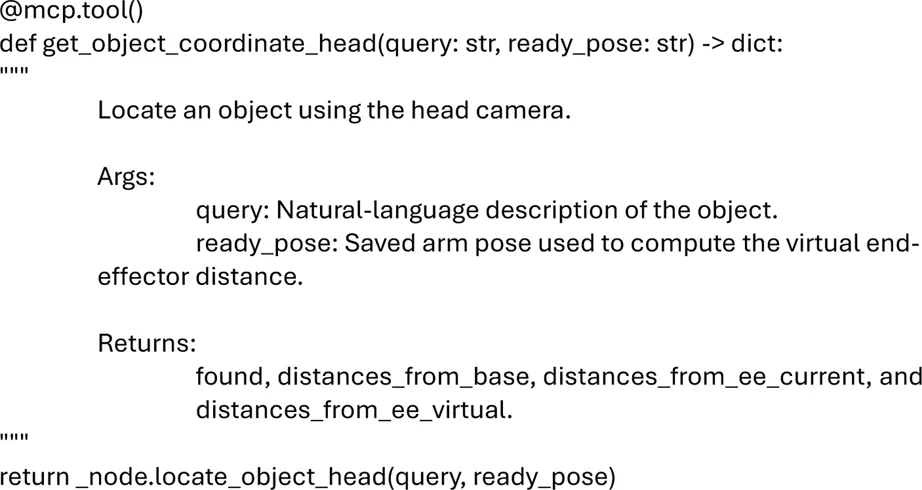
Example MCP tool exposing object localization.

The 28 MCP tools are organized into six functional categories in this study, including navigation, motion and manipulation, communication and recording, speech and transcription, retrieval, and perception. The following paragraphs describe the brief description of tools in each category. Navigation tools, such as “navigation_to_location”, use the ROS2 navigation stack (Macenski et al., 2020) for goal-directed navigation. Short local base motions, such as “move_base” and “base_turn”, are performed through velocity command publication. These tools allow the agents to request spatial movement through MCP calls without directly managing the underlying navigation and base-control interfaces. Task-specific details, such as how named locations or pose targets are used during object delivery, are described in Section 5.4.

The motion and manipulation tools provide non-navigation robot movements. Motion planning for arms utilized MoveIt (Coleman et al., 2014) interfaces. These tools support arm preparation, end-effector alignment, vertical and horizontal arm motion, and object manipulation steps used in the physical task. Other motion tools control the head, gripper, torso, or short body movements through ROS 2 joint control, trajectory actions, or velocity commands. This separates navigation through the mobile base from local robot motion and manipulation.

The communication and recording tools connect the agent system to the patient and clinician web interfaces. Tools such as “write_to_patient”, “write_to_clinician”, “ask_patient”, and “ask_clinician” send messages to the web interfaces or collect user responses. Recording tools such as “start_recording” and “stop_recording” control browser-based recording sessions and return recording outputs such as audio file paths and timing information. These tools allow task agents to include user interaction and documentation steps as explicit tool calls.

The speech tools connect the agent system to both robot speech output and speech transcription. The “speak” tool uses the robot onboard speaker through the robot speech interface. Transcription tools use a separate backend Whisper server (Radford et al., 2022). The transcription tools convert recorded audio files, or multi-role recording sessions, into text that can be used by the agents during documentation or interaction tasks.

The retrieval tool supports the patient education task. Patient education PDF materials are ingested before runtime, divided into text chunks, embedded, and stored in a Supabase vector database (Supabase, 2023). At runtime, the “search_patient_education” tool embeds the query and retrieves relevant chunks from the “documents” table through the “match_documents” function. The retrieved chunks are returned to the agent as tool results and used to generate patient-facing educational responses.

The perception tools provide image inspection (e.g., “check_status”, “capture_frame”) and object grounding (e.g., “get_object_coordinate_head”, “get_object_coordinate_wrist”). Their routines, including backend grounding, segmentation, and depth projection, are described in Section 5.6.

### 5.4 Task agent development

We developed five healthcare tasks as separate task agents, including object delivery, documentation, patient visual monitoring, patient education, and medication reminder. Each task agent uses the same shared MCP tools described in Section 5.3 and the same execution loop described in Section 5.5. When designing a task agent, a clinician writes a short description of the task and a set of phases for the agent to follow. The main agent reads the task description to understand which agent can handle a certain request from a user. The phase description is a critical part that defines how the task agent performs the task. It controls which tools the agent uses and in what order, what the agent reports and in what format, what the VLM pays attention to, and who the agent interacts with and how. Through this process, a clinician composes a unique task design for each situation while using only the available shared tools. The following subsections describe each task through its phase flow, and each subsection refers to a figure that lists the phases in order and the tools called within each phase.

#### 5.4.1 Object delivery agent

The object delivery agent handles any request to fetch an item from the supply table and deliver it to the patient. The agent drives the base, finds the requested object, grasps it, navigates to the delivery location, and releases the object. Figure 6 shows the phase flow and the tools that each phase uses.

**FIGURE 6:**
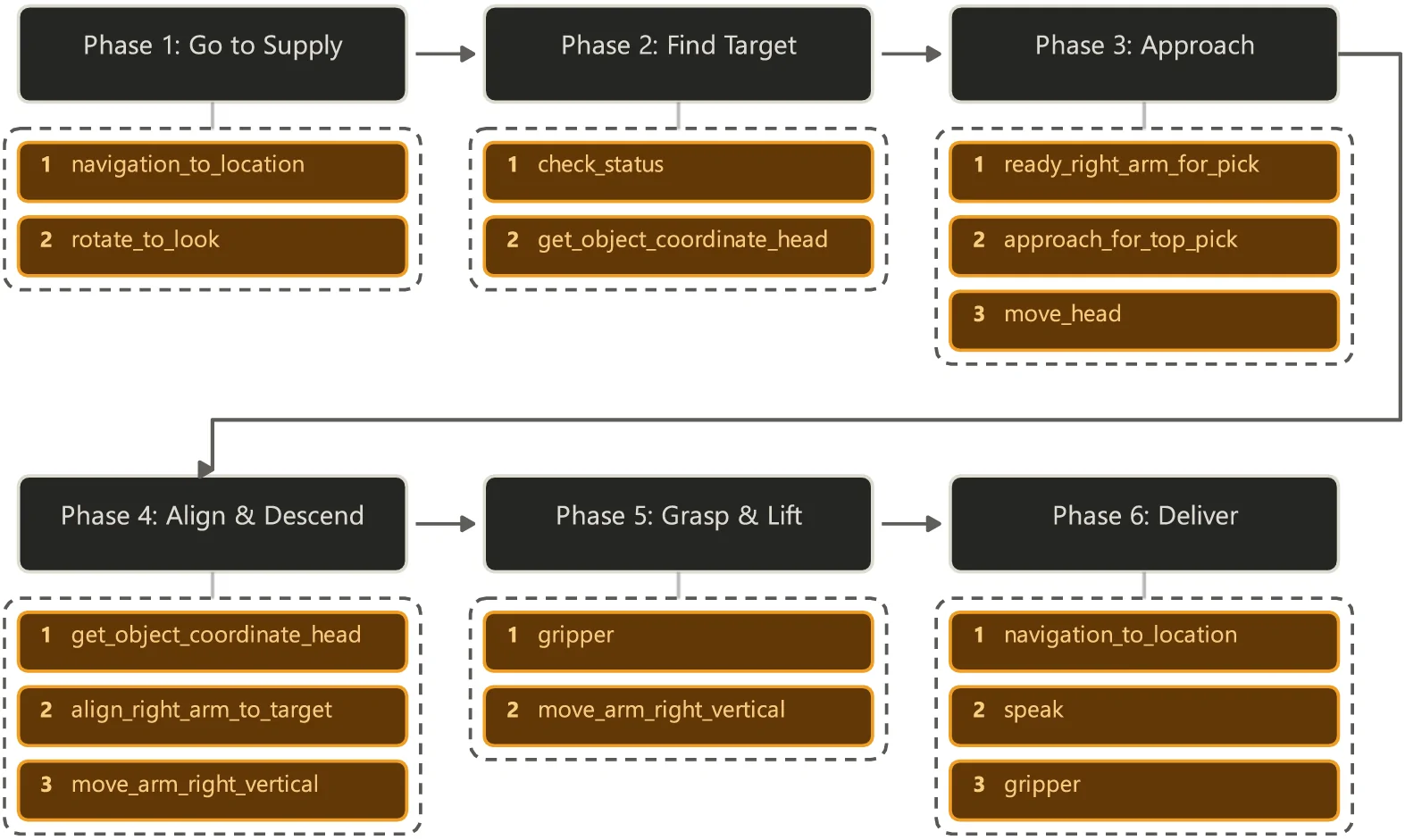
Object delivery task agent phases and tools.

In the first phase, the agent calls “navigation_to_location” and “rotate_to_look”. “navigation_to_location” reads a stored named location, which holds a base position and heading, and drives the mobile base to that location through the navigation stack (Macenski et al., 2020). “rotate_to_look” reads a stored look target and turns the robot to face it. The clinician defines these locations when designing the task, so the robot works from known positions rather than reasoning about a room it does not know, which keeps its behavior predictable and controllable.

In the second phase, the agent calls “check_status” and “get_object_coordinate_head”. “check_status” returns the current camera frames as images, which the execution loop adds as visual input for the next reasoning step, and the agent confirms that the requested object is visible. “get_object_coordinate_head” localizes the named object from the head camera and returns its position as x, y, and z distances measured from the top pick pose that the arm will later take, which the tool computes from the stored ready pose by forward kinematics. The grounding, segmentation, and depth projection inside this tool are described in Section 5.6.

In the third phase, the agent calls “ready_right_arm_for_pick”, “approach_for_top_pick”, and “move_head”. “ready_right_arm_for_pick” moves the right arm to the stored ready pose above the table for a top pick. “approach_for_top_pick” reads the measured distances and drives the base with omnidirectional straight-line motion, forward or backward and left or right, to reduce the horizontal distance between the pick pose and the object. “move_head” tilts the head camera down and keeps the object in view.

In the fourth phase, the agent calls “get_object_coordinate_head” again, “align_right_arm_to_target”, and “move_arm_right_vertical”. The agent re-measures the object position after the approach, because the base reaches its target less accurately than the arm, and the second measurement gives the arm an accurate target before it moves. “align_right_arm_to_target” moves the gripper horizontally over the object, and “move_arm_right_vertical” lowers the gripper onto it. These arm moves follow straight-line trajectories that MoveIt (Coleman et al., 2014) plans.

In the fifth phase, the agent calls “gripper” to close on the object and “move_arm_right_vertical” to lift it. In the sixth phase, the agent calls “navigation_to_location” to move to the delivery location, “speak” to tell the patient, and “gripper” to release the object. “speak” sends text to the robot onboard speaker.

The agent performs each manipulation as a deterministic motion that follows from perception and planning rather than from a learned visuomotor policy such as a vision-language-action (VLA) (Brohan et al., 2023; Kim M. J. et al., 2024) model. The object position comes from the perception tool, the base and arm move follow from that position, and a motion planner creates the arm trajectories that the system checks before motion. We chose this approach because a clinical environment requires behaviors that are designed for each situation and that a person can inspect and control. A learned end-to-end policy acts as a black box that does not allow the customization of robot behavior for different situations, nor the fine-tuning and debugging that this requires.

#### 5.4.2 Documentation agent

The documentation agent records a conversation between a clinician and a patient, transcribes both sides, and returns a summary to the clinician. While mature ambient clinical documentation tools already exist, this study does not incorporate those tools and implements the task with our own tools that can later be replaced by such mature solutions. The agent runs in two parts, a start part that begins the recording and a stop part that ends it and produces the summary. The agent selects the part from its prior history, since it begins recording when no recording is active and ends recording when one is active. Figure 7 shows the phase flow and the tools that each phase uses.

**FIGURE 7:**
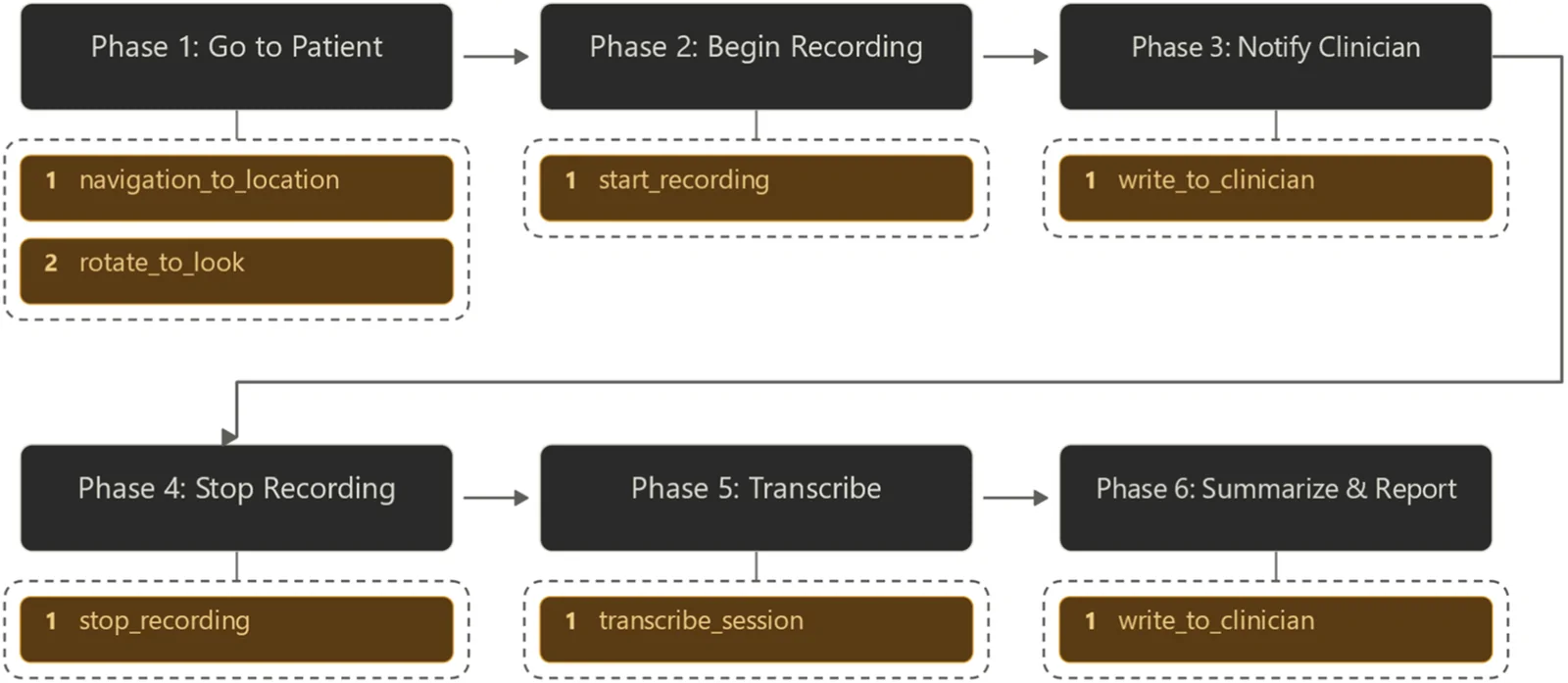
Documentation task agent phases and tools.

In the start part the agent moves to the patient with “navigation_to_location” and “rotate_to_look”, calls “start_recording” to begin audio capture on the patient and clinician pages, and calls “write_to_clinician” to report that recording has begun. “write_to_clinician” sends text to the clinician web page. In the stop part the agent calls “stop_recording” to end capture and obtain the audio, “transcribe_session” to transcribe both sides and interleave them by time, and “write_to_clinician” to send a summary followed by the full transcript. “transcribe_session” returns the combined transcript as a tool result, which the execution loop adds to the message history, and the agent writes the clinical summary from that transcript without adding facts that the transcript does not contain.

#### 5.4.3 Visual monitoring report agent

The patient visual monitoring agent observes the patient through the head camera and returns a structured report to the clinician. As in the documentation task, mature tools exist for this kind of monitoring, but this study limits the task to a simpler form where the agent’s VLM reads a fixed number of camera frames, analyzes them, and fills a fixed report template. This task is intended for the clinician, and the agent declines it when a patient requests it, as instructed in its prompt with a line such as “Only a clinician may request patient monitoring; if a patient requests it, decline and explain that this task is restricted to clinicians.” Figure 8 shows the phase flow and the tools that each phase uses.

**FIGURE 8:**
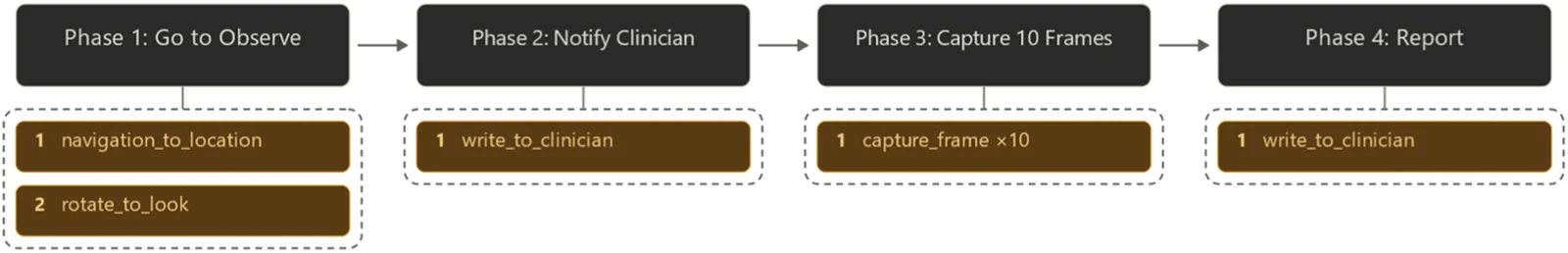
Patient visual monitoring task agent phases and tools.

In the first phase the agent moves to the observation location with “navigation_to_location” and “rotate_to_look”. In the second phase it calls “write_to_clinician” to report that monitoring has begun. In the third phase it calls “capture_frame” ten times and describes the patient after each frame. “capture_frame” returns a single frame from one selected camera as an image, and the execution loop adds each frame as visual input, and the agent observes the patient across the ten frames. In the fourth phase it calls “write_to_clinician” to send a report that covers posture, movement, visible distress, equipment, and anomalies.

#### 5.4.4 Patient education agent

The patient education agent answers a medical question from the patient using the hospital’s vetted patient education materials. The agent grounds every answer in these materials and does not use its own background knowledge. As in the documentation and monitoring tasks, a mature clinical question-answering system could fill this role, and this study instead implements the task as a basic retrieval-augmented generation (RAG) pipeline over a small set of vetted documents. The current materials are a heart transplant medication guide (Nebraska Medicine, 2024), a pre-heart-transplant education guide (WVU Medicine, Transplant Alliance, 2020), and a sickle cell disease fact sheet (National Heart, Lung, and Blood Institute, 2022). Figure 9 shows the phase flow and the tools that each phase uses.

**FIGURE 9:**
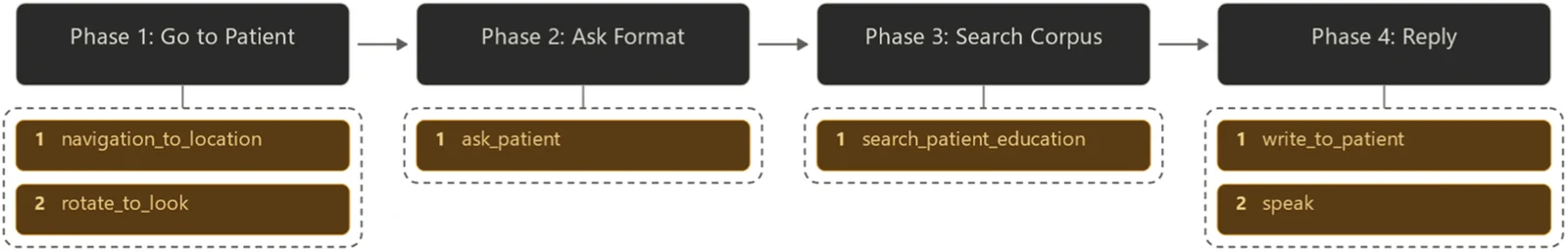
Patient education task agent phases and tools.

In the first phase the agent moves to the patient with “navigation_to_location” and “rotate_to_look”. In the second phase it calls “ask_patient” to ask whether the patient wants the answer spoken or shown on the screen. “ask_patient” sends text to the patient web page and waits for a reply. In the third phase it calls “search_patient_education” to retrieve the passages that match the question. In the fourth phase it calls “write_to_patient” or “speak” to give the answer, and it lists the sources when it writes the answer. “write_to_patient” sends text to the patient web page.

The patient education materials are prepared before runtime. We read each PDF page by page, divide the text into chunks of 1,000 characters with 200 characters of overlap, embed each chunk with the OpenAI text-embedding-3-small (OpenAI, 2025) model, and store the embeddings and metadata in a Supabase vector database (Supabase, 2023). At runtime “search_patient_education” embeds the question with the same model, calls the Supabase “match_documents” function, and returns the five closest chunks as a tool result. The agent writes the answer from these chunks.

#### 5.4.5 Medication reminder agent

The medication reminder agent alerts the patient about a scheduled event such as a medication, meal, vital sign check, activity, rest, or sleep cue. A separate scheduler process creates the timed trigger for this task and posts it to the main agent inbox, and the main agent sends the trigger to this agent through the same dispatch process used for other input commands. In a deployment this trigger would come from the hospital’s scheduling or electronic health record system, and the scheduler in this study stands in for that source. The agent itself only delivers the reminder to the patient, rather than deciding what or when to remind. The scheduler is described in Section 5.7. Figure 10 shows the phase flow and the tools that each phase uses.

**FIGURE 10:**
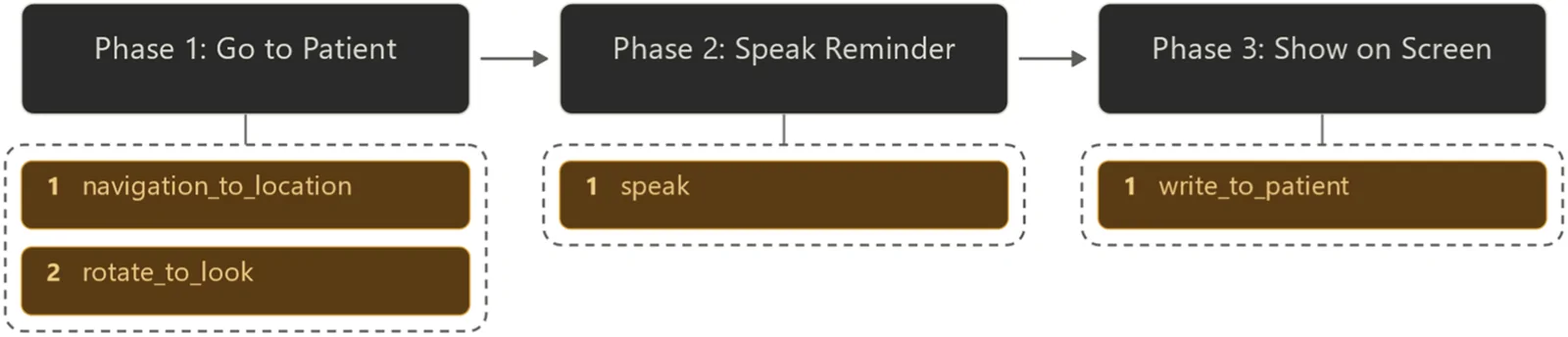
Medication reminder task agent phases and tools.

In the first phase the agent moves to the patient with “navigation_to_location” and “rotate_to_look”. In the second phase it calls “speak” to say the reminder out loud. In the third phase it calls “write_to_patient” to show the same reminder on the patient screen. The reminder text comes from the schedule entry in the triggering message, and the agent says and shows that text in plain language.

### 5.5 Iterative tool execution by agents

We implemented task execution as repeated interaction between the agent and the MCP tools, where the result of one tool becomes the basis for the next action. The execution loop sends the current message history and the available MCP tools to the VLM agent. When the agent returns tool calls, the loop runs the corresponding MCP tools and adds their results to the message history, and the agent uses the updated history to decide the next action. When a tool result includes image paths, the loop loads those images and adds them as visual input after the tool result, and the agent uses the image together with the text command and the previous tool results. The agent repeats this cycle until it returns a response without a tool call. Figure 11 shows this loop as pseudo code.

**FIGURE 11:**
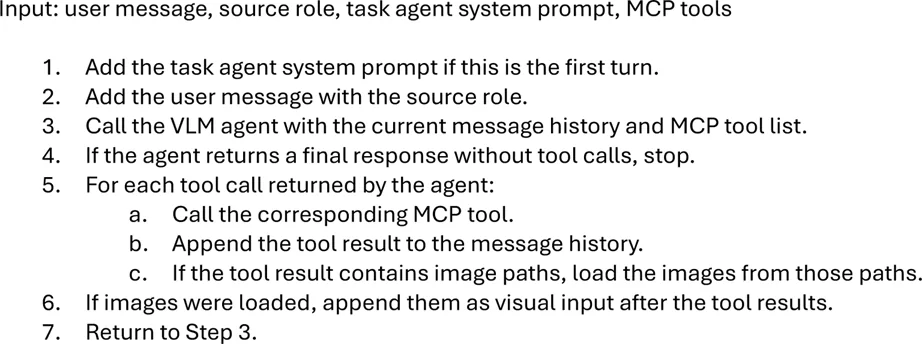
Pseudo code for iterative agent-tool execution.

### 5.6 Explicit visual perception and object grounding

The perception tools convert camera input into either an image that the agent inspects or a measured object position that the manipulation tools use. We implemented two perception routines for these two needs. The first routine captures an image for visual checking. The second routine localizes a requested object from RGB-D input through visual grounding, segmentation, and depth projection. Both routines run as MCP tools, and perception is an explicit tool call rather than a hidden step inside the agent. Figure 12 shows the two routines as separate paths.

**FIGURE 12:**
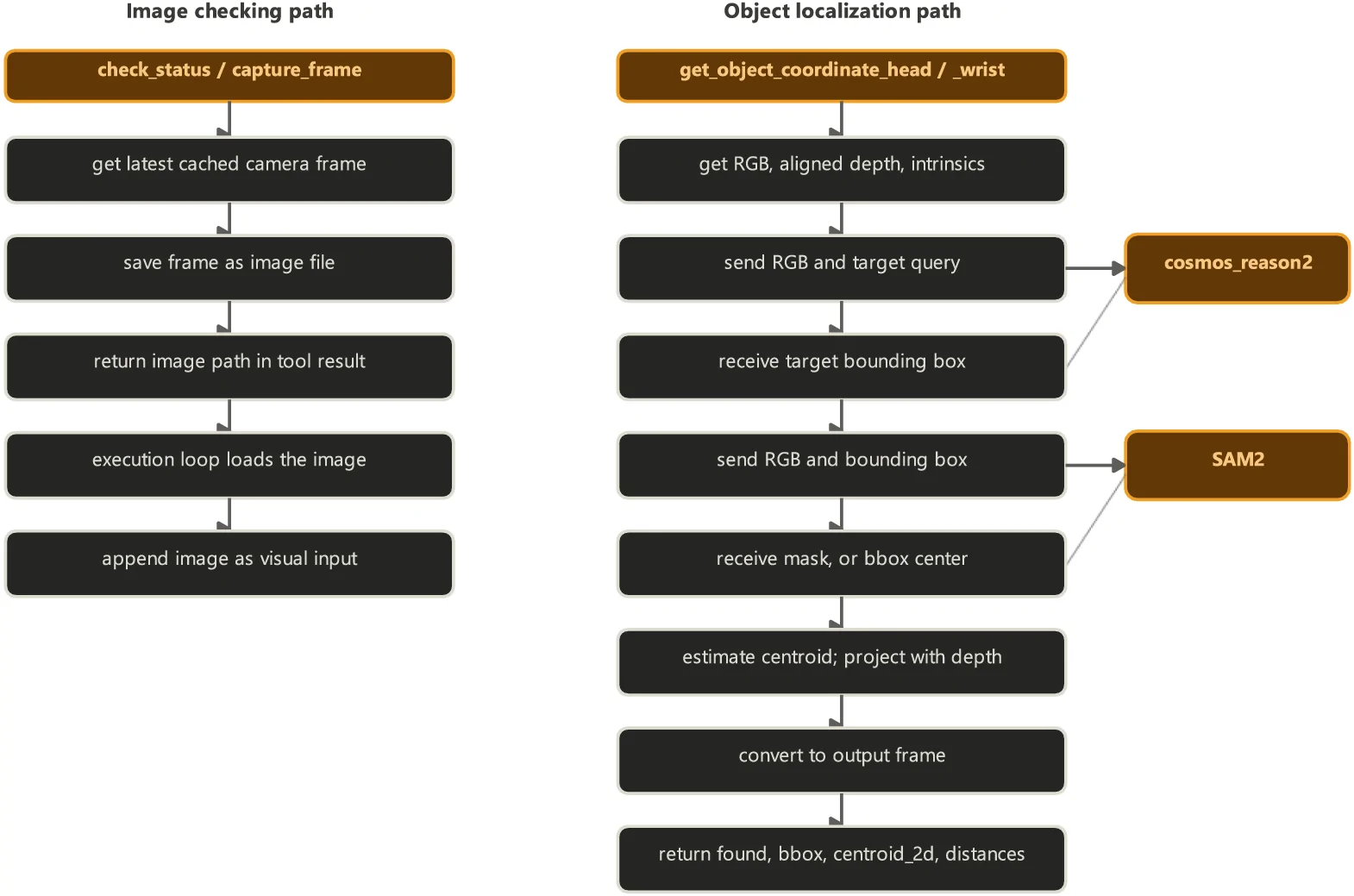
Object grounding and image checking perception paths.

The image checking routine gives the agent the current camera view. “check_status” returns the latest cached frames from all status cameras, and “capture_frame” returns the latest cached frame from one selected camera. Both save the frames as image files and return the image paths in the tool result. The execution loop loads the returned images and adds them as visual input for the next reasoning step, as described in Section 5.5. The agent then inspects the frames and describes what the camera shows. The visual monitoring task uses this routine to observe the patient across repeated frames, and other tasks use it to confirm the scene before acting.

The object localization routine returns a measured position for a named object. “get_object_coordinate_head” and “get_object_coordinate_wrist” get the RGB frame, the aligned depth frame, and the camera intrinsics from the selected camera. The routine sends the RGB frame and the target query to the Cosmos Reason2 visual grounding server, which returns a bounding box for the object. We use a VLM specialized in grounding here because a general VLM recognizes the named object but places its bounding box inaccurately, and an inaccurate box gives an inaccurate position. The Cosmos Reason2 model (NVIDIA, 2026) is specialized in visual grounding and returns a bounding box that locates the object well enough for the segmentation and depth steps to estimate its position. The routine sends the RGB frame and the bounding box to the SAM2 segmentation (Ravi et al., 2024) server, which returns a segmentation mask. The routine finds the target centroid from the mask, and it uses the bounding box center when no mask is returned. The routine reads the aligned depth around the centroid and uses the camera intrinsics to estimate the three-dimensional position of the object. The routine converts this position into the output frame used by the calling tool. The head camera tool returns two distances, the distance from the robot base to the target and the distance from the end effector to the target when the arm is in the top pick pose, while the wrist camera tool returns the same two distances. Whichever camera sees the target, the tool provides the same information in the same format, so the robot can move to that position accurately for picking. The routine returns whether the target was found, the bounding box of the target, the centroid of the target in the image, and these distance fields. The object delivery task uses the head camera tool, and it uses these distance fields for approach and alignment, as described in Section 5.4.1. The grounding Cosmos Reason2 and segmentation SAM2 servers run as backend services, which Section 5.7 describes.

### 5.7 Backend services

Several MCP tools and runtime processes rely on separate backend services. Object localization uses the cosmos reason2 (NVIDIA, 2026) grounding server and the SAM2 segmentation (Ravi et al., 2024) server (Section 5.6). The patient education retrieval tool uses a Supabase vector database (Supabase, 2023) (Section 5.4.4), and the transcription tools use a Whisper server (Radford et al., 2022) (Section 5.4.2). The scheduler runs as a separate process that reads the patient schedule file, checks the local clock every minute, and posts matching entries to the main agent inbox with the scheduler role, and the main agent handles them through its normal dispatch process (Section 5.4.5).

## 6 Demonstration and evaluation

This section demonstrates the feasibility of the proposed RHA architecture as the reasoning core for multifunctional healthcare robots. The case study performs the five tasks created during the development phase in Section 5 in a real patient room without human participants, focusing on the orchestration capabilities of the architecture. The demonstration examines whether a user can adapt a task agent to a given situation by editing the task phases, whether the main agent understands the user intent and calls the right task agent, and whether each task agent completes all of its phases as the task design specifies. We also compared three language models on these tasks, the commercial gpt-5.4-mini and the open-weight qwen3.5-122b-a10b and qwen3.5-35b-a3b. The experiment was conducted in an empty patient room in the Innovation Design Unit (IDU) at Nebraska Medicine (NM), with a TIAGo Pro robot. As shown in Figure 3, the room contains a hospital bed with bedside medical devices, an overhead monitor, and a wall-mounted control panel. The commercial model was accessed through the OpenAI API, and the two open-weight models were accessed through OpenRouter.

Before the experiment, we built a two-dimensional map of the room with gmapping. On this map, we obtained the locations that the tasks need by clicking each point in RViz2 and saving the point published on the “/clicked_point” topic. These locations are listed in Table 2. “navigation_to_location” uses a saved location to move the robot to a position and heading, and “rotate_to_look” uses a look target to turn the robot toward a point without moving. The task-relevant goal poses were predefined to maintain predictable and controllable robot behavior in the safety-critical patient-room environment. In a clinical deployment, such locations could be specified or approved by clinical staff according to the room layout, patient condition, and clinical workflow. These named locations define only the navigation goals, while Nav2 generates the paths at runtime using the robot’s current localization and costmap.

**TABLE 2:** Saved locations used by the navigation tools.

Location name	Used by	x	y	yaw
room_center	navigation_to_location	1.20	−0.09	−2.05
patient_observation	navigation_to_location	1.26	−0.67	1.59
patient_chat	navigation_to_location	−0.13	1.24	−0.09
patient_delivery	navigation_to_location	0.26	0.57	1.17
supply_table	rotate_to_look	0.31	−0.69	-
Patient	rotate_to_look	2.03	1.78	-
exit_door	rotate_to_look	3.84	−0.79	-
Delivery	rotate_to_look	0.78	1.30	-

Finally, we modified the final phases of the object delivery task for this room. In the development version, the task ends by driving to a fixed delivery position and releasing the object there. In this room, the robot instead turns toward the delivery table with “rotate_to_look”, checks the view with the head camera, measures the position of the table, approaches it, and places the object on it. We made this change to reflect the clinician’s intent of delivering the object onto the table in this room. The final phase now calls “move_head”, “rotate_to_look”, “check_status”, “get_object_coordinate_head”, “approach_for_top_pick”, “gripper”, and “speak”, which changes the task from 15 tool calls to 19 tool calls.

For the object delivery task, we issued the command “deliver the medication bottle to the patient. It is a white bottle on the cabinet.” We added the color and location of the bottle to this command because the grounding model often failed to identify the bottle when the command omitted the color feature, and naming the bottle as white made the identification reliable. The main agent received this command and, as its first tool call, invoked the object delivery task agent. From this point the object delivery agent ran the task through its own tool calls, which the following figures show.

Figure 3 shows the robot performing phase 1, 2, and part of 3, moving to the saved room center, finding the target medication bottle, and preparing its arm for the pick, in both an image and the matching RViz2 view. The robot drives from its start position to room_center (phase 1, tool call 1, “navigation_to_location”) and turns toward the supply table (phase 1, tool call 2, “rotate_to_look”), shown in Figure 3a with the saved locations from Table 2, and in Figure 3b as the planned path on the costmap in RViz2. The robot then confirms that the white bottle is visible in the head camera (phase 2, tool call 3, “check_status”) and locates it (phase 2, tool call 4, “get_object_coordinate_head”), which Figure 13 shows in detail. The robot then moves the right arm to the stored top pick pose (phase 3, tool call 5, “ready_right_arm_for_pick”), shown in Figure 3c and in RViz2 in Figure 3d.

**FIGURE 13:**
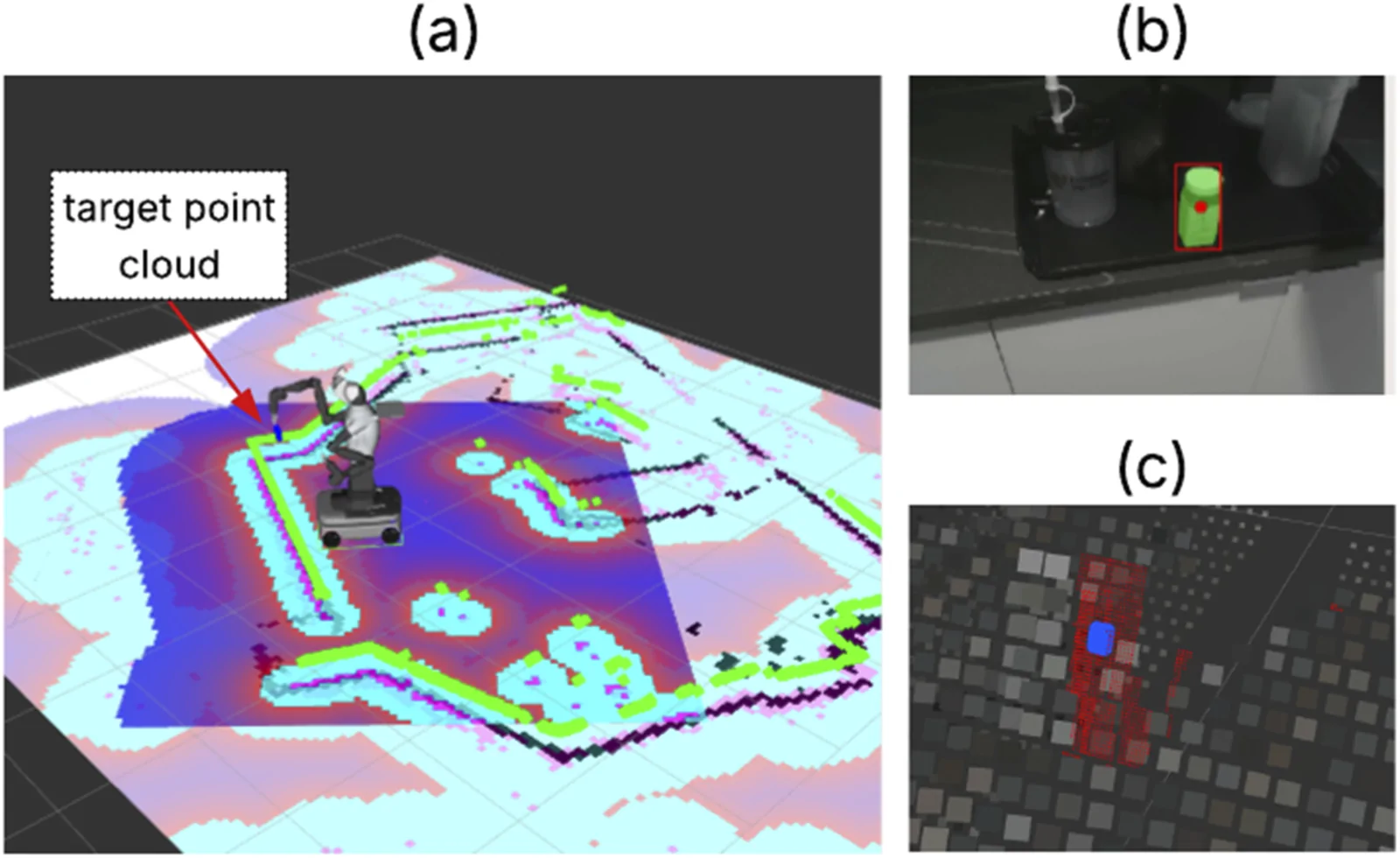
RHA localizing target with bounding box, mask, and centroid (tool call 4). **(a)** RHA at room_center in the ready pose on the costmap in RViz2, with the target point cloud of the bottle on the supply table in front of it. **(b)** Head camera RGB image with the bounding box from Cosmos Reason2, the segmentation mask from SAM2, and the centroid computed from the mask on the target bottle. **(c)** Close-up of the point cloud showing the target mask points and the centroid.

Figure 13 shows how the robot locates the white bottle inside the localization tool (tool call 4, “get_object_coordinate_head”), through the three internal steps described in Section 5.6. The tool sends the head camera RGB frame and the object query to the Cosmos Reason2 grounding server and receives a bounding box, then sends the frame and the box to the SAM2 server and receives a segmentation mask. Figure 13b shows the target image with the bounding box, the mask, and the centroid computed from the mask. Figure 13a shows the target point cloud in RViz2, and Figure 13c shows the target mask and its centroid in the point cloud.

Figure 14 shows the robot picking up the white bottle and placing it on the delivery table (the rest of phase 3 through phase 6). The robot drives the base until the pick pose is above the target (phase 3, tool call 6, “approach_for_top_pick”) and tilts the head to keep the target in view (phase 3, tool call 7, “move_head”), shown in Figure 14a. The robot re-measures the target position after the approach (phase 4, tool call 8, “get_object_coordinate_head”), moves the gripper over the bottle (phase 4, tool call 9, “align_right_arm_to_target”), lowers the gripper onto it (phase 4, tool call 10, “move_arm_right_vertical” down), closes the gripper (phase 5, tool call 11, “gripper” close), and lifts the bottle (phase 5, tool call 12, “move_arm_right_vertical” up), shown in Figure 14b. For this room, phase 6 places the bottle on the delivery table. The robot tilts the head (phase 6, tool call 13, “move_head”), turns toward the delivery table (phase 6, tool call 14, “rotate_to_look”), confirms the table in the head camera (phase 6, tool call 15, “check_status”), measures the table position (phase 6, tool call 16, “get_object_coordinate_head”), and drives the base to the table (phase 6, tool call 17, “approach_for_top_pick”), shown in Figure 14c. The robot then releases the bottle onto the table (phase 6, tool call 18, “gripper” open) and tells the patient (phase 6, tool call 19, “speak”), shown in Figure 14d.

**FIGURE 14:**
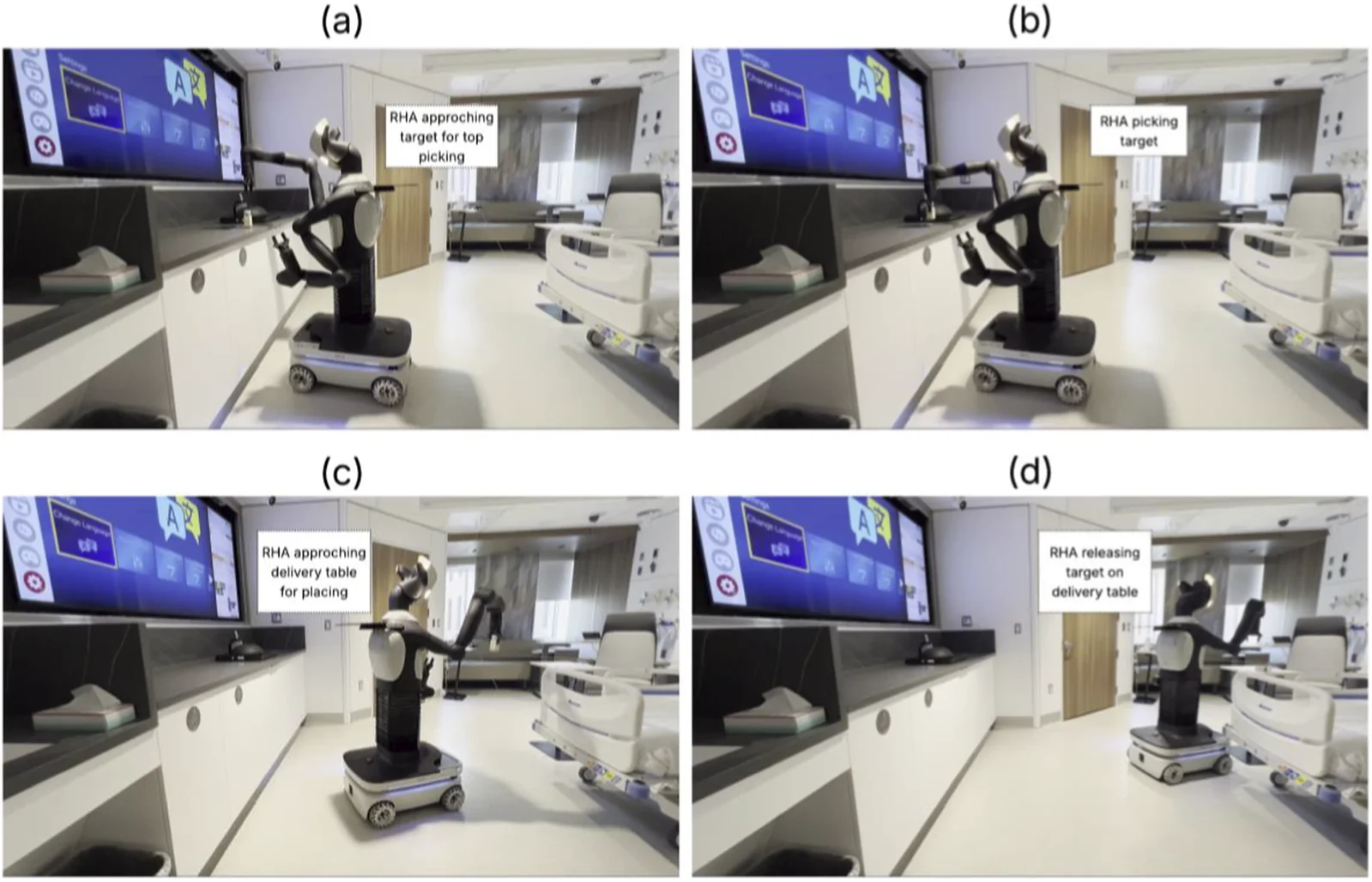
RHA picking bottle and placing it on the delivery table (phase 3 to phase 6 and tool calls 6–19). **(a)** RHA at the supply table approaching the target for the top pick (tool calls 6–7). **(b)** RHA lowering the gripper and picking the bottle (tool calls 8–12). **(c)** RHA holding the bottle and approaching the delivery table for placing (tool calls 13–17). **(d)** RHA releasing the bottle on the delivery table (tool calls 18–19).

The other four tasks each move the robot to a task location and then call the tools defined in their natural-language phases without manipulation. So instead of describing each one in full detail as we did for the object delivery task, we briefly summarize the process of each below. In the patient education task, a patient asked a medical question, and the agent moved to the patient chat location (“navigation_to_location”, “rotate_to_look”), asked whether to speak or show the answer (“ask_patient”), retrieved matching passages from the education corpus (“search_patient_education”), and read the grounded answer aloud (“speak”). In the demonstration the patient asked about recovery after a heart transplant, and the agent spoke a short answer drawn from the corpus, covering the hospital stay, incision discomfort and pain control, the recovery timeline, and the driving and lifting restrictions. This task speaks the answer without checking that a person is present, which we note as a limitation of the current design. In the patient visual monitoring task, as shown in Figure 15, a user issued “perform patient monitoring”, and the agent moved to the patient observation location (“navigation_to_location”, “rotate_to_look”), notified the clinician (“write_to_clinician”), captured ten head camera frames and described each (“capture_frame”), and sent a filled report covering posture, movement, distress, equipment, and anomalies (“write_to_clinician”), as shown in the bottom of Figure 15. In the documentation task, the agent moved to the patient chat location, began recording (“start_recording”), and after the author read a clinician-patient conversation aloud, ended recording, transcribed both sides (“stop_recording”, “transcribe_session”), and sent a summary with the full transcript to the clinician (“write_to_clinician”). In the medication reminder task, a scheduler process posted a timed trigger to the main agent, which dispatched it to the reminder agent, and the agent moved to the patient chat location and delivered the reminder by voice and on screen (“speak”, “write_to_patient”).

**FIGURE 15:**
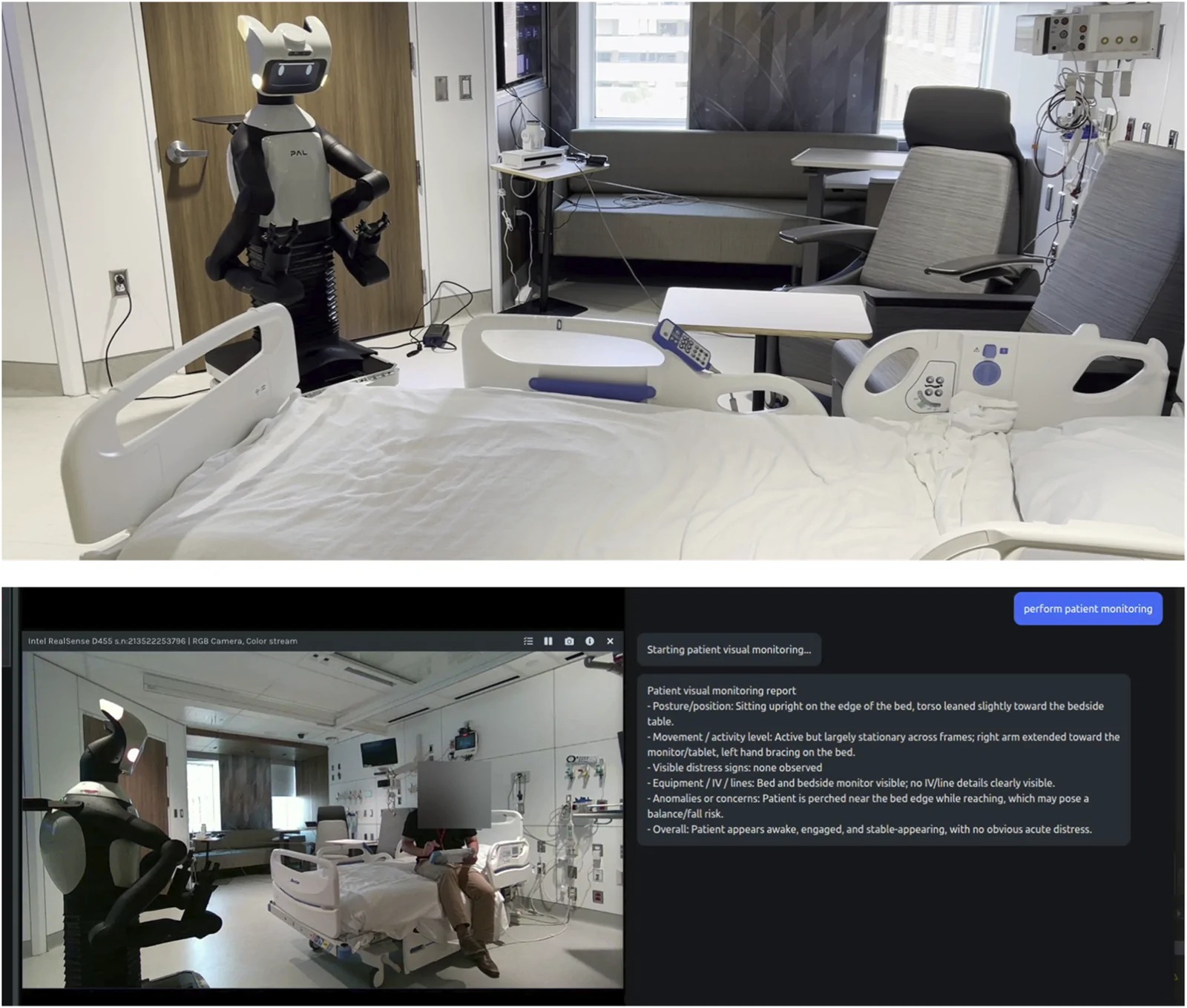
RHA performing the patient education task (top) and the patient visual monitoring task (bottom).

We evaluated whether each agent was dispatched correctly and ran its tool sequence to completion, rather than the quality of its retrieval or monitoring output. The other four task agents use comparatively simple execution structures, even when a task includes repeated tool calls such as frame capture. Each of these four tasks was also run 10 times per model, and they completed across all models and all trials, so we report the model comparison in detail only for the object delivery task. This task runs six phases and nineteen tool calls, and it is the only task whose length exposes differences between models.

We compared three reasoning models on this task, gpt-5.4-mini, qwen3.5-122b-a10b, and qwen3.5-35b-a3b. Each model ran ten trials with the same prompt and the same scene. Table 3 reports the results. qwen3.5-122b-a10b completed all six phases in every trial and produced a correct multi-step summary. gpt-5.4-mini completed nine of ten trials. In one trial it skipped the approach step in the final phase and opened the gripper before reaching the delivery point, and it still reported the delivery as successful. qwen3.5-35b-a3b completed six of ten trials. Its failures were consistent, since it stopped at the third phase near “move_head”, skipped all later phases, and reported the delivery as successful.

**TABLE 3:** Object delivery results across three reasoning models (10 trials each).

Model	Complete success	Description on phases	Average LLM time/calls	Average task time
qwen3.5-122b-a10b	10/10	All 6 phases completed in every trial	41.6s/20 calls	155.3 s
gpt-5.4-mini	9/10	6 phases completed; 1 trial skipped the approach step in phase 6, opened the gripper early, and reported a hallucinated success	66.5s/20 calls	164.2 s
qwen3.5-35b-a3b	6/10	4 trials stopped at phase 3 near “move_head”, skipped all later phases, and reported a hallucinated success	48.4s/20 calls	165.7 s

The clearest difference between models is the false report of success. In these cases the agent does not complete the task but tells the user that the delivery succeeded. qwen3.5-122b-a10b produced no such report, gpt-5.4-mini produced one, and qwen3.5-35b-a3b produced four. In a medication delivery setting a false report of success is more dangerous than a visible failure, so this failure matters more than the completion count alone.

The two failure modes share a pattern. Both models dropped required tool calls in the middle or late part of the sequence rather than encountering a reported failure within an individual navigation, perception, or manipulation tool. The smaller model stopped near Phase 3 in four trials, whereas gpt-5.4-mini skipped the approach step in Phase 6 in one trial. In both cases, the model stopped issuing the required tool calls and generated a final success response. Because the current runtime terminates execution when the model returns a response without a tool call, it treated this premature response as normal task completion and did not compare the executed steps against the phase specification. These observations identify the immediate execution-level failure mechanism, but the experiments do not establish the internal model-level cause of the omitted calls. A larger study across models, sequence lengths, and runtime configurations would be required to determine whether the failures arise from model capability, accumulated context, tool-calling behavior, or their interaction.

The response time is averaged over the successful trials. The language model time was 41.6 s for qwen3.5-122b-a10b, 48.4 s for qwen3.5-35b-a3b, and 66.5 s for gpt-5.4-mini, across about twenty calls in each trial. We do not read these differences as a property of model size, since the models run through a routing service and the serving path affects the response time. The task time was similar across the three models at 155–166 s, because the robot motion time dominates the total and varies between trials. We therefore treat the language model time as the cleaner comparison and the task time as a secondary measure.

Across these results the models separate on reliability rather than on speed. The speed difference is small once the robot motion is included, while the difference in completion and in false reports is large. Reliability is therefore the main driver for model selection in this system.

## 7 Discussion

This study tested a VLM based multiagent architecture for a multifunctional RHA on real robot hardware. The main agent routed natural language requests to the appropriate task agent, and each task agent followed its phase specification through shared MCP tools. The five tasks covered object delivery, documentation, visual monitoring, patient education, and medication reminders. The object delivery task was also adapted to the patient room by editing its phase description, without changing the agent code. These results support the central claim of the paper that a single RHA architecture can support multiple healthcare tasks by allowing the authoring of task agents by assembling shared tools through natural language phase specifications. The proposed architecture provides task-level scalability rather than unrestricted capability-level scalability. Clinicians can create or modify task workflows through natural-language phase specifications when the required actions can be composed from the existing MCP tools. A task requiring a new physical, perceptual, communicational, or informational capability would still require a developer to implement and validate a new low-level tool. Once added to the shared MCP layer, however, that capability can be reused across multiple task agents without rebuilding each task-specific pipeline.

The comparison of VLMs showed that long tool call sequences remain difficult for the current system. The four non delivery tasks had simpler execution structures, even when they included repeated calls such as frame capture, and completed across all models and trials. Object delivery required about 20 calls across six phases, including navigation, perception, base adjustment, arm alignment, grasping, delivery, and reporting. The 122B Qwen model completed all 10 trials. The GPT mini model completed 9 of 10 trials and skipped one late approach step. The 35B Qwen model completed 6 of 10 trials and stopped near the middle of the sequence in four trials.

The most serious failure was a false success report. In these cases, the agent skipped part of the task and still reported that delivery was complete. This type of failure matters in healthcare robotics because the user may not immediately know that the requested task remains unfinished. The result identifies task state consistency across tool calls as a key issue for VLM agent robots performing long, multi step tasks.

The skipped steps and false success reports may reflect limitations of both the reasoning models and the current agent runtime. The current implementation remains a simple tool calling agent rather than a more sophisticated agent runtime. The current agents append tool results to the message history and generate the next tool call from that history. More robust execution would require explicit task state tracking, phase completion checks, reasoning traces carried across tool calls, and recovery behaviors such as replanning and retrying. More specifically, a future runtime could maintain a structured state representation of the VLM-generated phase plan, mark each phase as pending, active, completed, or failed, and permit a success report only after all required completion conditions have been verified. Tool results would update this state outside the conversational history, and an execution monitor would intercept a termination response, return any incomplete or failed state to the VLM, and request retrying or replanning before allowing the task to end. A Behavior Tree could serve as an optional supervisory layer for this purpose, while the VLM retains responsibility for interpreting natural-language task specifications and adapting the task plan.

The study has several limitations in scope. The evaluation focused on whether the proposed architecture could dispatch requests, follow task agent phase specifications, and use shared MCP tools across multiple healthcare tasks on a real robot. Clinical performance was outside the scope of this evaluation, including the quality of the generated documentation summary, monitoring report, patient education response, and patient interaction. The prototype RAG, reporting, and scheduling components were evaluated only as tool implementations within the architecture and should not be interpreted as validated clinical systems. The trials were conducted in a single unoccupied patient room without patients or clinical staff, and the task specifications, room locations, and phase structures were prepared before the experiment. The results therefore support structured task execution under predefined room and task conditions, rather than clinical performance or autonomous care decision making.

Future work should evaluate these runtime mechanisms across different VLMs, agent frameworks, SDKs, and tool-calling implementations. Such comparisons should treat the model-runtime combination, rather than the reasoning model alone, as the unit of evaluation. Another significant research direction is to enhance the situational awareness of the robot. The current system perceives the room mainly through a narrow head camera, which limits what the robot can judge about the patient room. In particular, patient-facing tasks should verify that the intended recipient is present and visible before delivering spoken or displayed information. Wider sensing through additional cameras or panoramic perception could let the robot account for more of the room and support dedicated safety checks. Object-grounding tools should also detect uncertain or ambiguous targets and request clarifying visual attributes or a new camera view rather than relying on the user to provide such details in advance. A representation of the room or ward with a digital twin could also give the clinician and the robot a shared reference for locations, objects, patient state, and task context, making task design and correction more interpretable than raw camera images or coordinates alone.

Finally, developing a practical RHA will require improved control and integration with existing clinical tools. The current motions are largely scripted, which keeps the robot behavior inspectable and limits the range of objects and situations it can handle. Learned policies, such as VLAs, may be added where scripted control is insufficient, but their use should be limited to parts of the task where the risk can be controlled, such as the final adjustment before grasping, while the phase structure and safety checks remain explicit. In such a hybrid design, the learned policy would operate within a bounded local action space, and its outcome would be returned to the phase-level runtime for explicit verification before the task proceeds. The documentation, reminder, and patient education tools used in this study are prototype tools for demonstrating the architecture. Integrating established clinical systems for these functions would make the architecture more capable while preserving the same agent and tool structure.

## 8 Conclusion

This paper presented the RHA architecture, a VLM based multiagent design in which a main agent routes a natural language request to one of several task agents, and each task agent executes a healthcare task through phase specifications over a shared set of tools. We deployed the architecture on a TIAGo Pro robot in a real patient room and demonstrated five healthcare tasks defined and requested in natural language. We also showed that the object delivery task could be adapted to the room by editing its phases without changing the agent code. The comparison of three VLMs showed that long tool call sequences can expose skipped steps and false success reports, especially in the object delivery task that required about 20 tool calls. These results support the architecture as a practical reasoning core for a multifunctional healthcare robot and provide a scalable basis for authoring and adapting task agents within the capability boundary of the shared tool layer. They also show the need for more robust agent runtimes, stronger situational awareness, improved manipulation control, and integration with established clinical tools.

## Data Availability

The original contributions presented in the study are included in the article/supplementary material, further inquiries can be directed to the corresponding author.
